# Top–Down Strategy Enabling Elastic Wood Nanocarbon Sponges with Wrinkled Multilayer Structure and High Compressive Strength for High‐Performance Compressible Supercapacitors

**DOI:** 10.1002/advs.202410397

**Published:** 2025-01-29

**Authors:** Song Wei, Caichao Wan, Xingong Li, Shanshan Jia, Ruwei Chen, Guanjie He, Yiqiang Wu

**Affiliations:** ^1^ College of Materials Science and Engineering Central South University of Forestry and Technology Changsha 410004 China; ^2^ Christopher Ingold Laboratory Department of Chemistry University College London London WC1H0A UK; ^3^ College of Forestry Sichuan Agricultural University Chengdu 611130 P. R. China

**Keywords:** compressible supercapacitor, high areal capacitance, high compressive strength, wood nanocarbon sponge, wrinkled multilayer structure

## Abstract

3D porous carbon electrodes have attracted significant attention for advancing compressible supercapacitors (SCs) in flexible electronics. The micro‐ and nanoscale architecture critically influences the mechanical and electrochemical performance of these electrodes. However, achieving a balance between high compressive strength, electrochemical stability, and cost‐effective sustainable production remains challenging. Here, a superelastic wood nanocarbon sponge (WNCS) with a wrinkled multilayer structure is developed via a facile “top–down” design on natural wood. These unique wrinkled nanolayers effectively alleviate stress concentration through elastic deformation, resulting in a high compressive strength of 580.6 kPa at 70% reversible strain. The significantly increased specific surface area, coupled with abundant micro‐mesopores and highly graphitized nanocarbon, promotes rapid ion/electron transport, enabling the WNCS to achieve an ultrahigh capacitance of 4.21 F cm^−2^ at 1 mA cm^−2^, along with excellent cyclic stability and rate capability. Furthermore, an asymmetric supercapacitor (ASC) using a WNCS anode and a NiCo‐layered double hydroxide cathode retains 71.8% of its initial capacitance after 1000 compression cycles and withstands stress up to 1.03 MPa without capacitance degradation. This sustainable, cost‐effective WNCS shows great promise for flexible, compressible, and wearable electrochemical energy systems.

## Graphical abstract

A sustainable “top–down” approach is created to transforming solid wood into a robust wood nanocarbon sponge (WNCS) with unique wrinkled nanolamellar configuration. WNCS achieves high compressive strength (580.6 kPa) and capacitance (4.21 F cm^−2^), outperforming most reported compressible electrode materials fabricated using polymer substrates and “bottom‐up” methodologies, positioning it as a leading material for compressible energy storage applications.

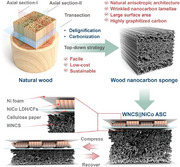

## 1 Introduction

The implementation of the global dual‐carbon strategy is leading to significant transformations in energy storage fields, shifting toward a more efficient, clean, and sustainable direction. Supercapacitors (SCs) have emerged as crucial energy storage devices for future sustainable energy needs, thanks to their high power density, long cycle life, fast charging and discharging capabilities, high safety, and flexible size adjustability.^[^ ^1^ ^]^ Given the explosive growth of portable and wearable electronic products, it is imperative to develop SCs with stable electrochemical performances and exceptional mechanical flexibility, including bendable, foldable one dimensional (1D) or two dimensional (2D) devices, and compressible elastic three dimensional (3D) devices.^[^ ^2^ ^]^ As the paramount component of compressible SCs, electrodes must not only guarantee feasible electrochemical performance but also demonstrate high compressive strength, outstanding elasticity, and durability.

**Figure 1:**
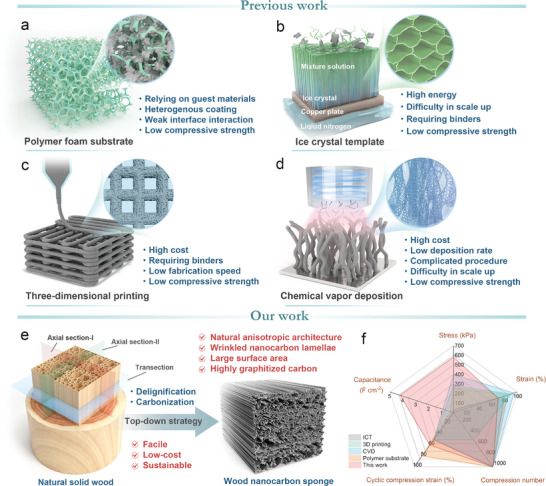
Comparison of our work and previous methods for fabricating compressible 3D electrodes. Previous works: a) Elastic polymer foam as the substrate for coating conductive materials. b) Ice crystal templating for fabricating elastic and conductive aerogels. c) 3D printing. d) CVD for fabricating graphene or CNTs aerogels. Our work: e) Top–down strategy for fabricating the highly compressible and conductive WNCS from natural wood. f) Radar plot showing the comprehensive performance of the WNCS compared to those of typical 3D compressible materials fabricated by other methods.

Many methods have been developed in recent years to create different types of 3D elastic porous materials for compressible electrodes, as summarized in **Figure** **1**a–d. Due to the inherent excellent elasticity, commercial melamine foam (MF) and polydimethylsiloxane (PDMS) as flexible scaffolds are being used to prepare compressible electrodes by coating various electroactive nanomaterials (Figure 1a).^[^ ^3^ ^]^ However, the weak interfacial bonding between guest and host is detrimental to the electrochemical stability under high compression strain and long cycles. Besides, direct carbonization of MF to obtain nitrogen‐doped carbon foam as an elastic electrode is also reported, but its low specific surface area results in low specific capacitance.^[^ ^4^ ^]^ Another feasible strategy for constructing compressible electrodes is “bottom‐up” nanofabrication, including ice crystal template (ICT), 3D printing, and chemical vapor deposition (CVD) methods, demonstrated in Figure 1b,c,d, respectively. During an ICT process, MXene, graphene, or carbon nanotubes (CNTs) are frequently incorporated into the slurry or gel precursors due to their benign 2D and 1D nanostructures and excellent performance.^[^ ^5^ ^]^ Directionally growing ice crystals act as templates to facilitate the assembly of these nanomaterials into aerogels with anisotropic porous structures, which can effectively relieve the loading stress concentration, granting them exceptional resilient properties in a specific direction.^[^ ^6^ ^]^ Due to the absence of strong interaction between graphene or MXene nanosheets, additional polymers are often required like polyurethane and polyimide, or nanocellulose and chitosan for enhancing the 3D molding property and mechanical strength, which further increases the process complication.^[^ ^7^ ^]^ This limitation is even more prominent in the 3D printing process since the “specific ink” requires more rigor on the components. CVD technology can convert carbon source gas directly into 3D CNT foam at high temperatures under catalytic conditions. It is beyond doubt that the extremely high cost and low production efficiency make it difficult to apply to practical energy products.

What is particularly significant is that, in the case of the majority of compressible materials mentioned above, while the maximum strain has been elevated to surpass 80%, the associated maximum stress remains relatively low (typically less than 200 kPa).^[^ ^8^ ^]^ This suggests that compressible devices constructed from these self‐supported elastic electrodes could potentially experience structural failure under heavier loads. Furthermore, current electroactive nanomaterials rely heavily on unsustainable, expensive resources and environmentally unfriendly processes. Hence, it remains a substantial challenge to persist in striking a delicate balance between outstanding mechanical robustness and electrochemical stability while achieving low‐cost and sustainable manufacturing of elastic electrode materials. Wood is a naturally porous material composed of biopolymers such as cellulose, hemicellulose, and lignin.^[^ ^9^ ^]^ Due to its abundant carbon content, widespread availability, low cost, renewability, and eco‐friendliness, it is widely regarded as a novel precursor material for fabricating carbon‐based SC electrodes. Enhanced electrochemical performance has been achieved by constructing electrochemical reactors within the aligned tubes of the wood carbon.^[^ ^10^ ^]^ The anisotropic porous structure and straight microchannels of the wood carbon facilitate rapid ion/electron transfer, while the open interconnected micropores and high specific surface area (SSA) allow for high‐mass loading, thereby improving the energy density of SCs. However, the dense, hard carbon layer structure of 3D wood carbon‐based thick electrodes restrict their application in flexible devices due to brittleness.

Herein, we propose a facile, low‐cost, and sustainable “top–down” strategy to develop solid wood‐derived 3D elastic electrodes for compressible SCs (Figure 1e). To corroborate the conception, balsa wood bulks were subjected to delignification and carbonization treatments, and wood nanocarbon sponges (WNCSs) with a wrinkled and multilayered architecture, superb mechanical compressibility and fatigue resistance were developed. As a promising electrode candidate for compressible SCs, WNCS obtains multiple benefits: i) The natural anisotropic architecture and wrinkled nanocarbon lamellae effectively reduce the compressive concentration and upgrade the stress strength. ii) The low density, large SSA, and abundant micro‐/mesopores of the nanoarchitecture render more efficient electrode‐electrolyte interfaces and shorten the migration distance of ion/electron. iii) The high graphitization degree realizes high surface charge density and promotes electron transfer. Consequently, WNCS exhibits a highly reversible compressive performance, including a high strain of 70% with a compressive strength of 580.6 kPa, and 1000 cycles at 60% strain. As a 3D compressible electrode for SC, WNCS delivers an ultra‐high specific area capacitance of 4.21 F cm^−2^ (176.3 F g^−1^, 11.7 F cm^−3^) at 1 mA cm^−2^ and can maintain 52.3% of the original capacitance at a large current density of 100 mA cm^−2^. The excellent mechanical properties and electrochemical performance of the WNCS lie far beyond those achievable with polymer substrate‐based and “bottom‐up” synthetic compressible electrodes (Figure 1f). A novel compressible quasi‐solid asymmetric supercapacitor (ASC) composed of WNCS anode and NiCo layered double hydroxide/carbon fibers (NiCo LDH/CFs) cathode achieves a high mechanical elasticity coupling with a high capacitance of 1.79 F cm^−2^ and a maximum energy density of 607.1 µWh cm^−2^. More impressively, the ASC device can sustain 71.8% of its original capacitance after 1000 cycles of compression and is at least amenable to stress up to 1.03 MPa without deteriorating the capacitance, which can meet the application requirements of various flexible and wearable electronics.

## 2 Results and Discussion

### 2.1 Preparation and Characterization of WNCS

**Figure 2:**
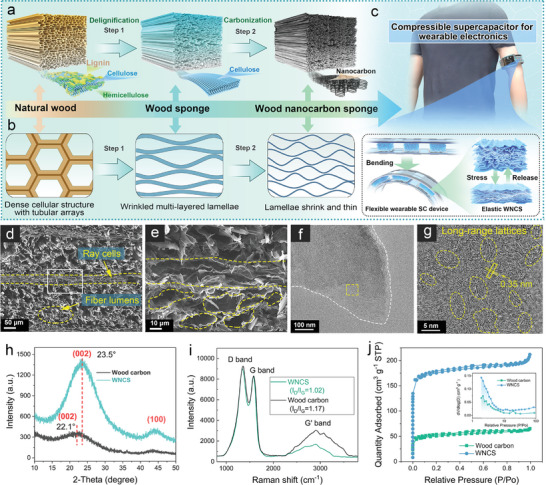
Fabrication and characterization of the WNCS. a) Schematic illustration of fabricating WNCS based on the “top–down” strategy and (b) the structural evolution of the microstructure from natural wood to WNCS. c) Schematic illustration of flexible and wearable SC devices with elastic WNCS electrode materials. d,e) SEM images and f,g) TEM images of WNCS at different magnifications. h) XRD pattern, i) Raman spectra, and j) N_2_ adsorption–desorption isotherms and pore distribution profiles of WNCS and wood carbon.

Natural wood has abundant honeycomb‐type vessels and lumen, in which the cell walls are composed of cellulose, hemicellulose, and lignin (Figure S1, Supporting Information). Specifically, cellulose nanofibrils are bound with hemicellulose and lignin via strong hydrogen/chemical bonds to construct dense and tough cellular layers, providing strong mechanical support for the axial growth of the tree. Therefore, the rigid cell wall structure of wood is prone to fracture during compression, which results in a highly plastic deformation (Figure S2, Supporting Information). The reasonable regulation of the architecture of wood through selectively removing the components from the cell wall can endow wood with excellent compressibility.^[^ ^11^ ^]^ As schematically illustrated in **Figure** **2**a, based on the modified method from Guan's group,^[^ ^12^ ^]^ we removed lignin and hemicellulose components from the wood cell wall by using NaOH and NaClO_2_ to obtain elastic wood sponges, in which cellulose fibers were retained. Subsequently, The WNCS was obtained by conducting a carbonization process at 1000 °C on wood sponges. This approach is facile and scalable, and the cubic shape of the wood remains intact throughout the preparation process (Figure S3a–d, Supporting Information). The as‐prepared WNCS displays a lightweight nature with a density of 42.6 mg cm^−3^ (Figure S3e, Supporting Information) and excellent compressibility (Figure S3f,g, Supporting Information). FTIR spectra (Figure S4, Supporting Information) show that the characteristic peaks of lignin and hemicellulose disappeared after chemical treatment, indicating the complete removal of lignin and hemicellulose. Moreover, XRD patterns (Figure S5, Supporting Information) justified that wood sponge possesses a higher degree of crystallinity than that of natural wood (55.5% vs 48.1%), due to the removal of amorphous lignin and hemicellulose.

Figure 2b presents the potential evolution of transection‐view cellular structures from natural wood to WNCS. Natural wood is mainly composed of countless vertically aligned hexagonal‐shaped tubes (fiber lumens) that are seamlessly connected (Figure S6, Supporting Information). After the removal of lignin and hemicellulose components in the cell wall, the cellulose microfibril aggregation layer is released to form a continuous wrinkled layered structure (Figure S7, Supporting Information). After carbonization, a thinner conductive carbonaceous multilayer structure is obtained. This distinctive anisotropic multilayer structure holds promise to endow wood‐based carbon materials with exceptional elasticity, rendering them highly suitable for use as electrode materials in compressible supercapacitors and flexible wearable electronic devices (Figure 2c).

Compared with the wood carbon, which remains the straight and large‐diameter fiber channels and ray cell structures of natural wood (Figure S8, Supporting Information), WNCS presents a similar wrinkled multilayers structure to wood sponge (Figure 2d). The wrinkled fiber tubular structures and the laminar structures of the ray cells of WNCS are shown in Figure 2e. Note that the thickness of these carbonaceous multilayers reduced to a finer nanoscale (Figure S9, Supporting Information), that is, nanolamellar carbon was obtained, indicating a significantly increased surface area. The view in axial section‐I presents wrinkled and aligned multilayers (Figure S10a, Supporting Information), whereas in axial section‐I displays a relatively flat surface with an anisotropic texture (Figure S10b, Supporting Information), further confirming the successful formation of nanolamellar structure. TEM images in Figure S11a (Supporting Information) and Figure 2f, respectively, show the carbon sheet morphologies of the wood carbon and WNCS with alternating dark and light edges, indicative of the multilayer structure. The high‐resolution TEM (HRTEM) images reflect that the wood carbon exhibits highly disordered carbon structures (Figure S11b, Supporting Information), while WNCS comprises disordered amorphous carbon and rich long‐range ordered graphite layers (marked by yellow circles, Figure 2g). It is measured that the spacing of lattice stripes in the ordered area of WNCS is 0.35 nm (Figure S12, Supporting Information), close to the interlayer spacing of graphene, indicating that the removal of amorphous lignin and hemicellulose in natural wood is conducive to improving the degree of graphitization of materials.^[^ ^13^ ^]^

The XRD pattern in Figure 2h shows two diffraction peaks corresponding to the (002) and (100) plane of carbon phases respectively. The wood carbon shows broader and weaker peak characteristics, reflecting the hard carbon phase from the carbonized natural wood. As to the WNCS, the peak intensity is much higher than that of the wood carbon sample, indicative of a higher graphitization degree, which can rationalize the appearance of abundant ordered graphite lattice stripes observed in HRTEM. Notably, compared to the peak center position of 22.1° for the wood carbon (002) plane, the peak center position of WNCS moves to 23.5°, indicating a smaller mean layer spacing. As shown in Figure 2i, Raman spectra present two characteristic peaks defined as G and D bands were observed at the absorption bands of 1576–1581 and 1338–1356 cm^−1^, respectively, corresponding to the vibration of graphitized sp^2^ carbon and the scattering of disorder/defects present in sp^2^ carbon. The I_D_/I_G_ of WNCS is 1.02, which is smaller than that of wood carbon (1.17), suggesting a lower defect density. In other words, the WNCS derived from wood cellulose with higher crystallinity features a higher degree of graphitization. In addition, a G’ band peak, which is composed of multiple Lorentz peaks, was observed at the absorption band of 2230–3625 cm^−1^, reflecting the multilayered carbon nanostructure.

Nitrogen adsorption tests were conducted to investigate the micro‐nano pore structure of the samples. The wood sponge exhibits increased mesopores and macropores compared to natural wood and a higher specific area (Figures S13 and S14, Supporting Information). This is attributed to substantial nanogaps arising from the decomposition of the dense cell wall into multiple layers during delignification. As shown in Figure 2j, wood carbon and WNCS both show the IV‐type isotherm classified by IUPAC. At relatively low pressures (*P*/*P*_0_ < 0.05), the adsorption capacity increases rapidly, with a slight hysteresis loop occurring within the range of 0.5 < *P*/*P*_0_ < 0.9, indicating the presence of abundant micropores and mesopores. The nitrogen adsorption capacity of WNCS is much higher than that of wood carbon, suggesting a richer micro‐nano pore structure, which is also confirmed by the pore distribution curves in the inset of Figure2j. As a result, the BET SSA and pore volume of WNCS are as high as 601.7 m^2^ g^−1^ and 0.328 cm^3^ g^−1^, respectively (Figure S14 and Table S1, Supporting Information), 3.5 and 3.2 times higher than that of wood carbon. In addition, the WNCS also shows a smaller average diameter of 2.179 nm, which further implicates the formation of abundant micropores and mesopores. The larger SSA and wrinkled multilayer structures give rise to rapid diffusion and adsorption of electrolyte ions and create more pathways for fast electron transportation.

### 2.2 Compressive Mechanical Property of WNCS

**Figure 3:**
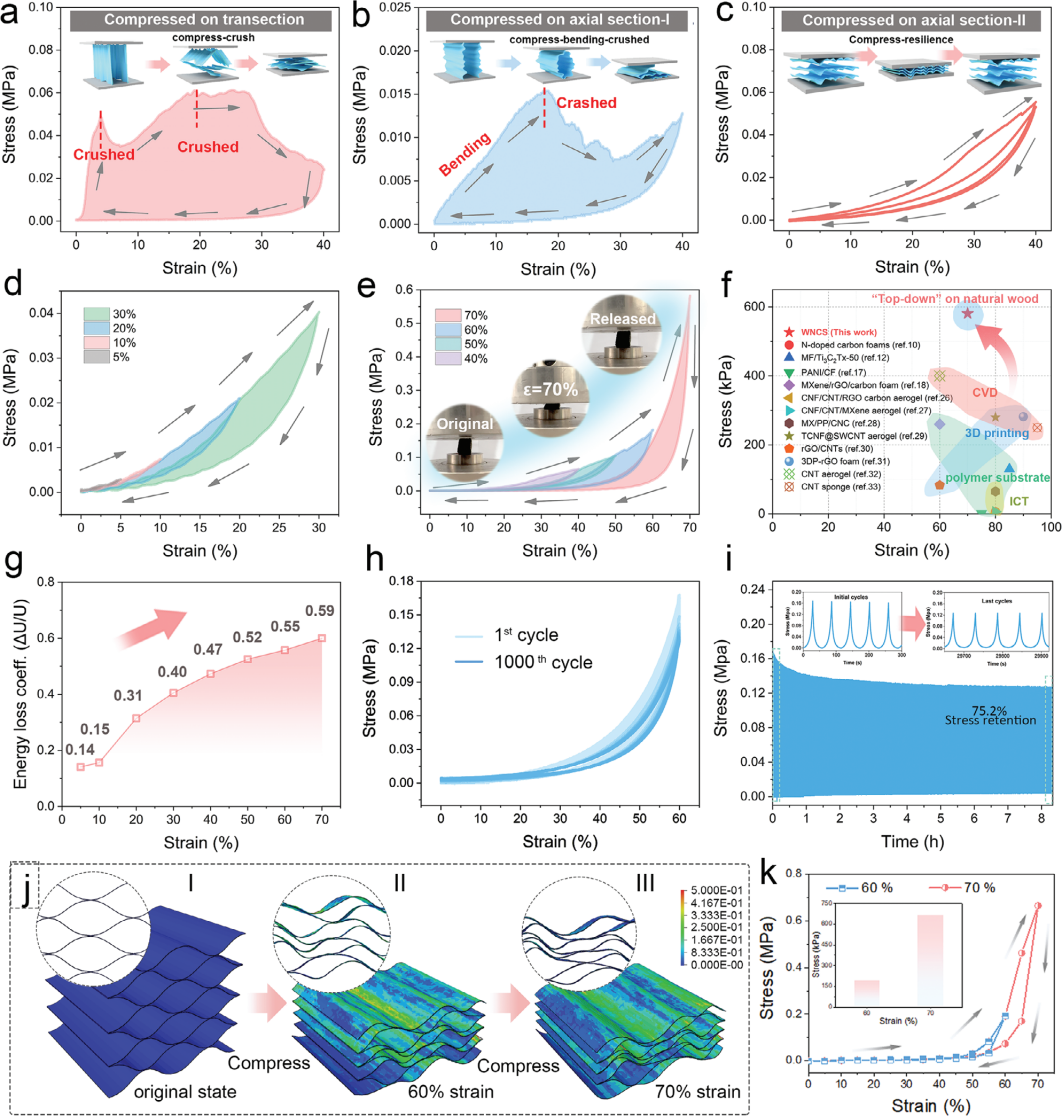
a–c) Compressive mechanical properties of the WNCS. *σ*–*ε* curves of (a) transection, b) axial section‐I, and (c) axial section‐II. d,e) Compressive *σ–ε* curves of axial section‐II at various strain levels from 5% to 70%. f) Comparison of the maximum resilient strain and stress of WNCS with other elastic 3D monolithic electrodes fabricated from different materials via various methods. g) Energy loss coefficient. Fatigue resistance at 60% strain for 1000 cycles: h) *σ*–*ε* curves, i) *σ–t* curves. j) FE simulation of the compression process in the axial section‐II of the WNCS at 60% and 70% strain. The circular area shows the corresponding morphologies from the 2D front view of the 3D models. k) *σ*–*ε* curves of the FE simulation at 60% and 70% strain for WNCS.

Compression tests were conducted to investigate the mechanical property and fatigue resistance of the wrinkled multilayer structure since they are crucial prerequisites for practical compressible electrodes. Considering that the wood connaturally features an anisotropic mechanical property, the compression tests on different sections of the samples were carried out. **Figure** **3**a–c records the stress–strain (*σ–ε*) curves of WNCS at the transection, axial section‐I, and axial section‐II in the range of 0–40% strains, respectively. It is worth mentioning that not only the aligned straight channels of wood itself lead to the anisotropy of its structure, but also the orientation of cellulose fibers in its cell wall is the main reason for its mechanical anisotropy (Figure S15, Supporting Information).^[^ ^9^ ^]^

Figure S16 (Supporting Information) shows the schematic illustrations of the compression processes in various directions of WNCS. For the compression *σ–ε* curves on the transection shown in Figure 3a, the stress increases dramatically at the strain range of 0–3.9%, and then a cliff descent appears from 3.9% to 7.3% strain, which reveals that the compression on the transection surface of WNCS is subjected to a large yield force, indicative of the rigidity of the wrinkle nanocarbon layers. As the compression continues to higher strains, a period of slow rising and falling stress appears, which is caused by the lateral stacking between the fracture blocks and the secondary rupture. Figure 3b shows another structural fragmentation behavior of the compression on axial section‐I. When the stress is gradually increased in the axial section‐I direction, the wrinkled nanolayer can undergo bending strain to a certain extent, and the structural fracture will not occur immediately. The stress increases until the strain reaches 18% and begins to decrease sharply, demonstrating that the collapse occurs at the weak interface bonding of the aligned nanofibers in the wrinkled nanocarbon layers, which leads to the collapse of the whole structure of WNCS. Besides, due to the lateral stacking between the fractured carbon sheets, a slight stress rise occurred in the process after 30% strain. Figure 3c shows the *σ–ε* curves after two cycles of axial section‐II compression, which exhibits a perfect crescent shape, indicating that the peculiar wrinkled multilayer structure of WNCS has excellent elasticity and damage resistance. In particular, the energy loss coefficient after the first circle compression test was 0.47, much lower than those of the other two compressions on transection (0.94) and axial section‐I (0.81) (Figure S17, Supporting Information). In addition, due to the structural activation effect caused by the compression of the first circle, the energy loss of the second circle is significantly reduced to 0.25, indicative of the excellent compression‐rebound property of WNCS.

The *σ–ε* curves of WNCS in different strain ranges were further tested. As shown in Figure 3d,e, the *σ–ε* plots composed of smoothly compressed and released curves in 5–70% strains exhibit a typical crescent shape, which can be separated into three distinct stages. In the range of 0% < ε < 30% (Figure 3d), the stress increases linearly with the strain, indicative of the elastic bending of carbon layers (region I). Subsequently, there is a plateau emerges within the 30% < ε < 50% range (region II), suggesting that the wrinkled multilayer structure starts to be compressed (Figure 3e). As the compressive strain increases to 70%, the stress increases dramatically (region III), resulting in large‐scale structural densification of WNCS. The inset digital images in Figure 3e and Figure S18 (Supporting Information) display the compression test process at 70% strain, demonstrating a perfect shape and structure recovery of the WNCS. In addition, the wood sponge which features a similar wrinkled multilayer structure exhibits the closely analogous compression‐recovery behavior (Figure S19, Supporting Information). However, due to the thick cellulose cell wall layers and the rich hydrogen bonds between the layers, the wood sponge can withstand the compressive rebound range of 0–60% at most (Figure S20, Supporting Information). On the contrary, the *σ–ε* plot of the wood carbon (Figure S21, Supporting Information) suggests that it failed to recover after compressive deformation, showing a typical brittleness (Figure S22, Supporting Information).

Exhilaratingly, the as‐prepared WNCS delivered a high stress of 580.6 kPa under the max strain of 70%, substantially surpassing those of 3D monolithic electrodes fabricated by other approaches like polymer substrate,^[^ ^3^, ^4^, ^8^ ^]^ ICT,^[^ ^14^ ^]^ 3D printing,^[^ ^15^ ^]^ and CVD^[^ ^16^ ^]^ (Figure 3f; Table S2, Supporting Information). Such significant levels of compressive stress and strain indicate that the WNCS exhibits exceptional durability against crushing and impacts, making it highly suitable for use in wearable electronics across diverse and challenging environments. The energy loss coefficient (ELC) of the first compression‐release cycle test of WNCS under different strains is provided in Figure 3g. When the strain is less than 10%, the ELC is almost unchanged at ≈0.15. As the strain continues to increase, the ELC begins to increase linearly. Finally, this value reaches 0.59 at the strain of 70%, indicating its outstanding structural stability. In an aspect of fatigue resistance, WNCS can withstand 1000 times of long‐term compression at 60% strain, and the stress retention rate can still reach 75.2% (Figure 3h,i). The stress in all the recovery curves remains above 0, indicating that there is almost no loss of height. Moreover, no significant structural collapse or cracks were observed in the microstructure (Figure S25, Supporting Information). These outstanding results are undoubtedly attributed to the unique architecture of WNCS. The nanoscale and wrinkled carbon multilayers, derived from highly graphitized fiber lumens and ray cells, can effectively disperse the compression stress concentration through the interlocking and bending between the layers, thus withstanding the large loads and maintaining excellent elasticity.

The stress distribution within the unique nanoscale wrinkled nanolayers of WNCS and the compact tubular structure of wood‐derived carbon under compression in axial section‐II was further analyzed using finite element (FE) simulations. As shown in Figure 3j–I, the structure of the WNCS can be defined as a periodic lattice consisting of wrinkled nanolayers, while the wood carbon dispalys a hexagon‐like lattice (Figure S24, Supporting Information). The simulation results reveal that when the WNCS is subjected to 40% strain (Figure S25a, Supporting Information), the nanoscale wrinkled layers effectively dissipate stress concentrations through spatial slippage and inherent elastic strain, allowing the overall WNCS structure to undergo significant dimensional contraction without any loosening or fracture. As shown in Figure 3j‐II,j‐III, when the compression strain increases from 60% to 70%, a more densely packed arrangement of the wrinkled nanolayers is observed, with a remarkably uniform stress distribution on the surface of the nanolayers with an enlarged contact area. After releasing the loading force, the WNCS recovers to the original shape without significant breakage of wrinkled nanolayers (Figure S25b, Supporting Information). Note that slight deformation can be observed (2D front view in Figure S25b, Supporting Information), which is due to the plastic deformation in certain regions of the wrinkled nanolayers. In contrast, wood carbon shows severe structural rupture due to major stress concentration at the uneven junctions under 40% compression strain (Figure S24, Supporting Information). As shown in Figure 3k, the *σ*–*ε* curves of WNCS based on FE simulation demonstrate that during the reversible compression process at 70% strain, WNCS exhibits a peak stress of 666 kPa, which is more than three times the stress observed at 60% strain (192.2 kPa). This finding aligns closely with the *σ*–*ε* curves obtained from practical ccompression measrements.

### 2.3 Electrochemical Performances of WNCS

Elastic, conductive, and porous 3D foams with large SSA and excellent mechanical properties are kind of ideal materials for self‐supporting thick electrodes. All the CV curves of WNCS electrodes with different thicknesses vary from 0.8 to 5.2 mm and display a nearly quadrilateral shape at 5 mV s^−1^ in the potential window of −0.8–0 V versus Ag/AgCl (Figure S26a, Supporting Information), suggesting a rapid and efficient charge transfer on the electrode surface. The corresponding GCD plots recorded at 1 mA cm^−2^ (Figure S26b, Supporting Information) show that the charging and discharging potentials change linearly and the two curves are almost symmetrical, further signifying the efficient electric‐double‐layer (EDL) and fast ion transport. The capacitance of the WNCS electrode is significantly improved with increasing thickness from 0.8 to 3.6 mm (Figure S26c, Supporting Information). Note that further increasing the electrode thickness up to 5.2 mm was unable to boost the capacitance, and even sacrificed the volumetric capacitance and gravimetric capacitance. Larger electrode thickness may lengthen the electron transfer path and slow down the ion diffusion, resulting in parts of the micro‐nano network within the electrode that cannot participate in rapid charge storage, becoming a “dead volume”. EIS results also demonstrated this inference (Figure S27, Supporting Information).

**Figure 4:**
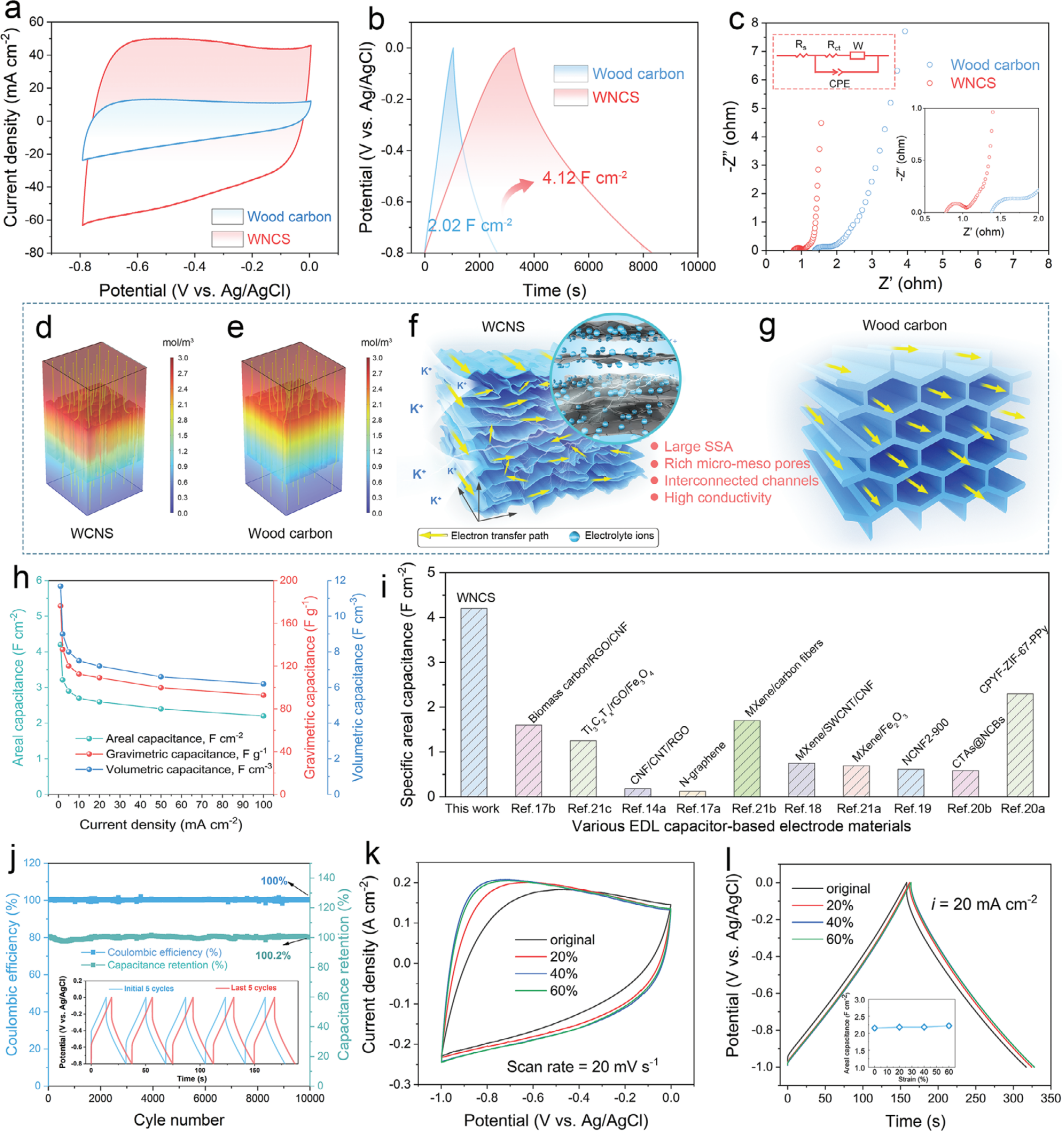
a–c) Electrochemical performance of the WNCS. Comparison of (a) CV and (b) GCD and (c) Nyquist curves of WNCS and wood carbon. d,e) Simulated K^+^ concentration distribution in (d) WNCS and (e) wood carbon, respectively. The boundary condition is set as 3 m KOH. f,g) Schematic illustration of ion and electron transfer mechanism within WNCS and wood carbon. h) Capacitances at different current densities. i) Comparison of the max specific areal capacitance of the WNCS with reported materials. j) Stability of WNCS for 10 000 cycles GCD test. k) CV curves under increasing compressive strains at a scan rate of 20 mV s^−1^. l) GCD curves under increasing compressive strains at a current density of 20 mA cm^−2^, the inset shows the corresponding capacitances.

The electrochemical properties of wood carbon and WNCS were compared at the same thickness (3.6 mm) to highlight the advantages of WNCS in the structure of electrochemical energy storage. Cyclic voltammetry (CV) measurement was carried out first to investigate the electrochemical behavior of WNCS and wood carbon (Figure S28, Supporting Information). Figure S27b (Supporting Information) shows the CV curves of WNCS at 5–100 mV s^−1^. When the scan rate is greater than 20 mV s^−1^, the CV closed ring gradually changes from a quasi‐rectangular shape to a spindle shape, which demonstrates a typical EDL behavior. As shown in **Figure** **4**a, WNCS exhibits a larger integral area of the cyclic voltammetry (CV) curve at 5 mV s^−1^, indicative of the enhanced specific capacitance. The galvanostatic charge–discharge (GCD) curves at various current densities are shown in Figure S29 (Supporting Information). As shown in Figure 4b, the WNCS delivers a remarkable specific area capacitance of 4.21 F cm^−2^ nearly 2 times that of wood carbon (2.02 F cm^−2^) at the current density of 1 mA cm^−2^. When the current density is increased by 50 times, the capacitance retention is as high as 57.0% (2.4 F cm^−2^) for WNCS, while wood carbon only retains 34.7% (0.52 F cm^−2^) of its original capacitance (Figures S29 and S30, Supporting Information), suggesting the excellent rate capability of WNCS. These results suggest the remarkable contribution of increased SSA and abundant pore structure to capacitance. Electrochemical impedance spectroscopy (EIS) was further conducted to reveal the kinetic of WNCS. Nyquist plots in Figure 4c show that WNCS features a smaller intercept of the horizontal axis and semicircle in low frequency compared with wood carbon. As a result, WNCS delivers an equivalent series resistance (*R*_s_) of 0.78 Ω and a charge transfer resistance (*R*_ct_) of 0.14 Ω, superior to that of wood carbon (*R*_s_ *=* 1.39 Ω, *R*_ct_ *=* 0.28 Ω), indicating fast charge transport due to the wrinkled nanocarbon layered whin continuous highly conductive pathways. According to the four‐point probe test result (Figure S31, Supporting Information), WNCS possesses a significantly higher electrical conductivity of 64.06 S m^−1^ than that of wood carbon (20.14 S m^−1^), further demonstrating an excellent capability of charge immigration, which can be attributed to a higher degree of graphitization.

To further reveal the unique merits of the wrinkled nanocarbon layers in contributing to the ions transfer, the FE analysis was carried out by COMSOL multiphysical field simulation. According to the SEM and BET characterization results, micromodels of WNCS and wood carbon electrodes were constructed, as shown in Figure 4d,e. From the comparison, such an interconnected porous structure composed of the ordered stacking of multiple wrinkled nanocarbon layers provides rich pathways for electrolyte ions to diffuse through the electrode, enabling the uniform distribution of electrolyte concentration gradient (Figure 4d). Inversely, due to the closed tubular structure with dense carbon wall, wood carbon presents a high concentration accumulation layer, resulting in a slow diffusion rate of electrolyte ions (Figure 4e).

As schematically illustration in Figure 4f, the outstanding electrochemical performance of the WNCS can be credited to the wrinkled nanolamellar configuration, which donates high SSA, significant pore volume, and excellent conductivity. i) The multilayer nanocarbon features open and interconnected spacing that facilitates the high‐flux adsorption of electrolyte ions onto the electrode surface (Figure 4d). ii) The large SSA and flourishing meso‐/micropores provide the adequate electrode‐electrolyte interface, promoting the ion diffusion throughout the electrode and enhancing additional EDL capacitance, which results in ultrahigh capacitance and excellent rate capability. iii) the highly graphitized nanocarbons derived from woody cellulose enable a faster and more efficient electron migration. On the contrary, for the wood carbon electrode, the poor porosity and enclosing cellular channels greatly limit the ion and electron transfer paths (Figure 4g).

Figure 4h shows that the WNCS exhibits an ultra‐high specific areal capacitance of 4.21 F cm^−2^ (11.7 F cm^−3^ for volumetric capacitance and 176.3 F g^−1^ for gravimetric capacitance) at the current density of 1 mA cm^−2^. Even when the current density is increased by 100 times, the capacitance retention rate can retain a high value of 52.6%. As shown in Figure 4i and Table S3 (Supporting Information), the max specific areal capacitance of the self‐supporting 3D WNCS electrode with a 3.6 mm thickness, fabricated by such a facile and low‐cost method, exceeds most of reported EDL capacitor‐type materials like graphene,^[^ ^17^ ^]^ CNT,^[^ ^14^, ^18^ ^]^ carbon nanofibers,^[^ ^19^ ^]^ and the emerging metal–organic framework (MOF)^[^ ^20^ ^]^ and MXene.^[^ ^21^ ^]^ Besides, in a 10 000 cycles GCD test at 50 mA cm^−2^, the coulombic efficiency of the WNCS is almost maintained at 100% during the cycling process, and the capacitance retention rate is up to 100.2% at the 10 000^th^ cycle (Figure 4j).

The electrical performance of WNCS was further investigated under various compressive states. Figure S32a (Supporting Information) presents the response curve of electrical resistance with well‐defined peaks during various compressions (0–60%). In the enlarged region under 5% strain (Figure S32b, Supporting Information), the resistance reduction and increased curves correspond to compression and release processes, respectively. Moreover, this resistance wave curve exhibits good stability and symmetry, even under the cycling compression of 60% strain (Figure S32c, Supporting Information). Note that the recovered resistance values are larger than the original, attributed to the energy loss of WNCS during the compression. Based on these repeated and stable resistance response signal peaks, the electrical resistances of WNCS at 0%, 20%, 40%, and 60% strains are 27.0, 16.0, 12.2, and 11.0 Ω, respectively (Figure S33, Supporting Information). Figure 4k displays the slightly expanding area of the CV curves recorded at the scan rate of 20 mV s^−1^ when the WNCS compressed under strains from 0% to 60%. The GCD curves at the current density of 20 mA cm^−2^ in Figure 4l exhibit a similar trend, with no obvious shape change, can be observed. Additionally, as shown in the inset of Figure 4l, the areal capacitances slightly increase with increasing strains, implying that compressive strains do not compromise the electrochemical performance of the WNCS. In other words, the WNCS can withstand high loads while maintaining superb capacitive properties. What's more, the proposed “top–down” strategy for natural wood includes merely delignification and carbonization processes, which are both cost‐effective (Table S5, Supporting Information) and sustainable. Consequently, such low‐cost WNCS with excellent mechanical elasticity and electrochemical properties is emerging as an ideal electrode material for compressible supercapacitors.

### 2.4 Electrochemical Performance and Mechanical Stability of WNCS||NiCo ASC

To further investigate the electrochemical performance of WNCS‐based compressible SCs, we prepared a typical NiCo LDH/CFs freestanding electrode as the cathode. NiCo LDH was synthesized by a rapid electrodeposition method on the carbon fiber substrate which is also a kind of biomass‐derived mechanically flexible and highly conductive material (Supplementary experimental section). SEM images show the well‐distributed honeycomb‐like NiCo LDH anchoring on CFs (Figure S34a–c, Supporting Information). The EDS mapping demonstrates the well‐distributed elements of O, Ni, and Co (Figure S33d, Supporting Information). Besides, the XRD pattern of NiCo LDH/CFs contains all diffraction peaks of NiCo LDH (PDF#33‐0429) without any impure peaks (Figure S35, Supporting Information). The CV curves of NiCo LDH/CFs electrode appear a couple of sensitive redox peaks in a potential window of −0.2–0.6 V (Figure S36a, Supporting Information), indicative of highly reversible Faradic reactions. The GCD curves demonstrate a typical battery‐type characteristic of distinct voltage plateaus (Figure S36b, Supporting Information), resulting in a maximum specific areal capacitance of 1.6 F cm^−2^ (Figure S36c, Supporting Information). The low internal resistance and charge transfer resistance of the NiCo LDH/CFs is conducive to fast and reversible redox reactions (Figure S36d, Supporting Information). Besides, the electrode can sustain 89.8% of the original capacitance even after 5000 cycles of GCD tests at 50 mA cm^−2^ (Figure S36e, Supporting Information). These results show that the as‐prepared NiCo LDF/CFs electrode is eligible to serve as the cathode for WNCS‐based compressible SCs.

**Figure 5:**
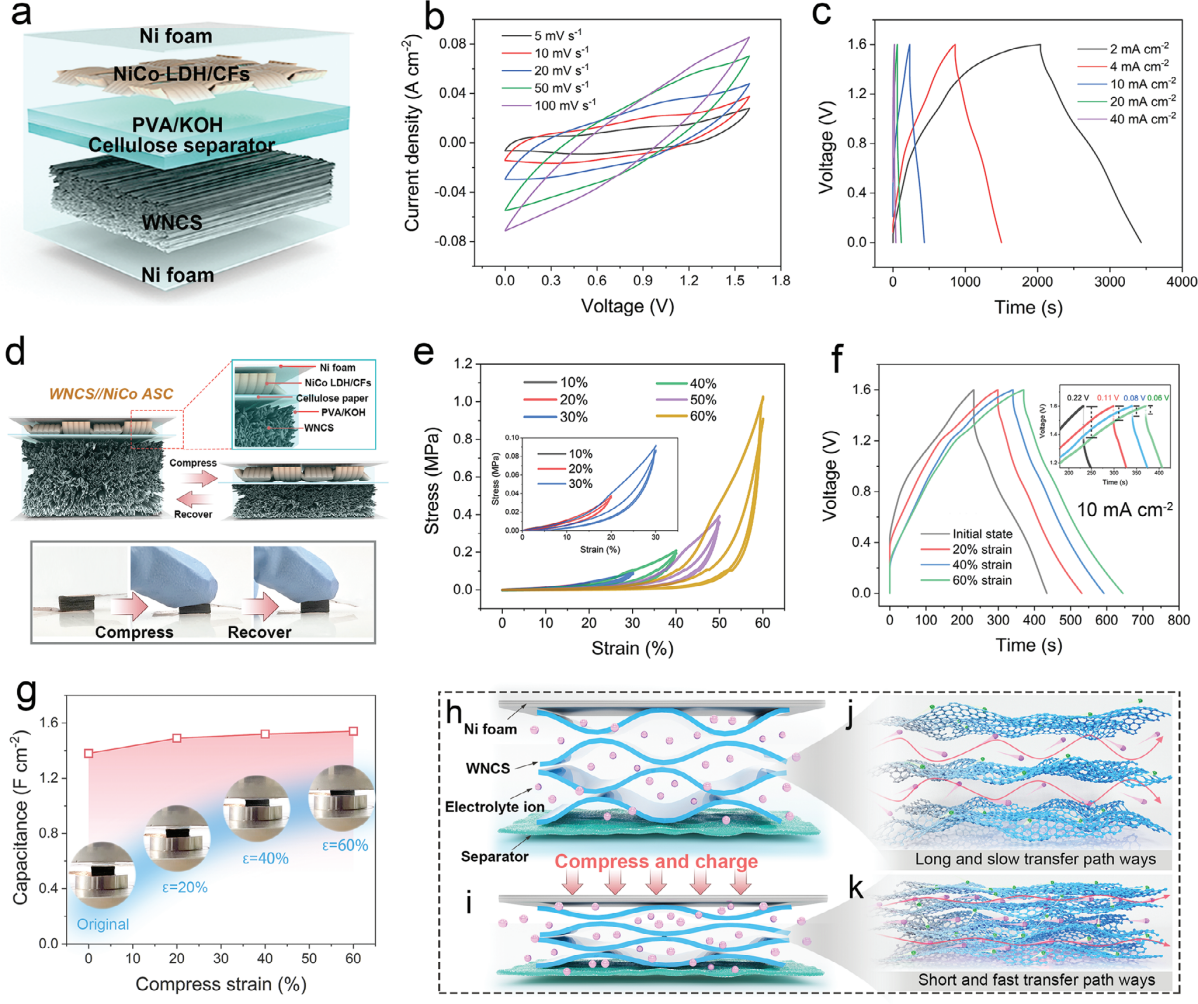
Electrochemical performance of the compressible WNCS||NiCo ASC. a) Schematic illustration of the sandwich‐like structure of the ASC device. b) CV curves at different scan rates. c) GCD curves at different current densities. d) Schematic diagram and digital images of the ASC before and after compression. e) Compressive *σ–ε* curves at the strain range of 10–60% (two consecutive test cycles under each strain condition. f) GCD curves and (g) specific areal capacitances under increasing compressive strains at a current density of 10 mA cm^−2^. h–k) Schematic diagram of electron/ion migration within the WNCS nanolamellae under original and compressed states.

As depicted in **Figure** **5**a and Figure S37 (Supporting Information), the WNCS anode, NiCo LDH/CFs cathode, cellulose paper as the separator, and PVA/KOH gel as the electrolyte were used to assemble the sandwich like WNCS||NiCo LDH/CFs quasi‐solid state asymmetric supercapacitor (WNCS||NiCo ASC). Figure S38 (Supporting Information) shows the integrated CV curves of the cathode and anode in the integrated potential window of −0.8–0.6 V at a scan rate of 5 mV s^−1^. It means that the WNCS||NiCo LDH/CFs ASC is promising to output as high as 1.6 V voltage. As a result, the CV curves of the device at various scan rates can be recorded in the anticipative wide voltage range of 0–1.6 V without decomposing the aqueous electrolyte. As shown in Figure 5b, the CV curve at 5 mV s^−1^ exhibits a similar fusiform shape accompanied by a pair of weak redox peaks. Increasing the scan rate even up to 100 mV s^−1^ does not change the shape of the CV curves pronouncedly, which is indicative of an excellent rate capability. GCD curves of the ASC in different current densities are provided in Figure 5c. It can be found that even under a low current density of 2 mA cm^−2^, the device can be still fully charged to 1.6 V, and no significant voltage drop emerges, indicating that the interface between the components of the device is well integrated. These GCD curves feature good symmetry, reflecting decent capacitive property and Coulombic efficiency. As a result, the ASC exhibits an ultra‐high maximum specific areal capacitance of 1.79 F cm^−2^ at 2 mA cm^−2^ and maintains a large value of 1.14 F cm^−2^ (63.7% retention) when increasing the current density by 10 times (Figure S39, Supporting Information). The capacitance can even reach 0.97 F cm^−2^ at 40 mA cm^−2^, a high retention rate of 54.1% is obtained. According to the GCD curves, the ASC can deliver a maximum of 607.1 µWh cm^−2^ at a power density of 1562 µW cm^−2^ and still retains the energy density of 94.6 µWh cm^−2^ while the power density is increased to 16.8 mW cm^−2^ (Figure S40, Supporting Information). These competitive values are superior to those of the wood or cellulose‐based 3D SCs,^[^ ^10^, ^14^, ^22^ ^]^ and other nonrenewable materials like graphene/CNT‐,^[^ ^23^ ^]^ and metal‐based^[^ ^21^, ^24^ ^]^ 3D SCs reported recently, as shown in Table S4 (Supporting Information). Moreover, the ASC also exhibits exceptional cycle stability with a 78.2% capacitance retention and a 91.7% coulombic efficiency at 20 mA cm^−2^ after cycling for 5000 times charge–discharge (Figure S41, Supporting Information).

SCs with excellent elastic deformation and resistance to compressive destruction have broad application prospects in flexible electronic devices. Since the volume of as‐prepared elastic WNCS almost occupies the whole device (Figure 5d), the WNCS||NiCo ASC can be squeezed easily and represent a full volume recovery when the pressure is released, exhibiting excellent compressibility. As shown in Figure 5e, the *σ–ε* curves of the ASC present a smooth crescent shape over the strain range of 10–60%, which demonstrates that the internal structure of the device is not damaged during the compression‐rebound process. Under 60% strain, the stress of the first and second compression circles are 1.03 and 0.91 Mpa, respectively, much higher than that of WNCS (0.580 Mpa), which can be attributed to the good penetration of PVA electrolyte. The large compressive stress means that the device can sustain greater loading before failure. The electrochemical performances of WNCS||NiCo ASC under 0%, 20%, 40%, and 60% compressive strains were investigated. As shown in Figure S42 (Supporting Information), the area enclosed by the CV curve at the scan rate of 10 mV s^−1^ extends slightly with increasing strain, but the shape remains almost unchanged, indicating the electrochemical stability of ASC. Moreover, GCD curves at the current density of 10 mA cm^−2^ present a consistent trend with CV curves (Figure 5f). It is observed that when the ASC is compressed from the original state to 60% strain, the discharge voltage drop is reduced from 0.22 to 0.06 V, suggesting that the internal resistance of ASC under compression is lower than that of the original (as shown in the inset of Figure 5f). As shown in Figure 5g, the capacitance of the ASC in the free state is 1.38 F cm^−2^, and it is increased to 1.49, 1.52, and 1.54 F cm^−2^ when the compressive strain is up to 20%, 40%, and 60%, respectively. In addition, EIS results further confirm that the ASC in a compressed state exhibits lower resistance and fast charge transfer (Figure S43, Supporting Information).

The mechanism of capacitance change of the compressed ASC device is relevant to the ion/electron transfer for the WNCS anode, which is regulated by the multilayer nanocarbon architecture that varies from external pressure. As schematically illustrated in Figure 5h,i, when the ASC is in a free state, certain regions of the WNCS anode show larger spacing between carbon nanolayers, resulting in a slow ion transfer behavior based on interlayer diffusion. The migration paths of electrons are also limited to the independent graphite lamellar surface. As shown in Figure 5j,k, as the carbon interlayer transfer channel is compressed by external force and narrows, the barrier to ion transport is greatly reduced, accelerating the superfluid migration of ions in these 2D channels with limited spacing.^[^ ^25^ ^]^ In addition, the electron transfer paths are richer as the increased contact area between nanocarbon layers, thus reducing the internal resistance of the ASC device.

**Figure 6:**
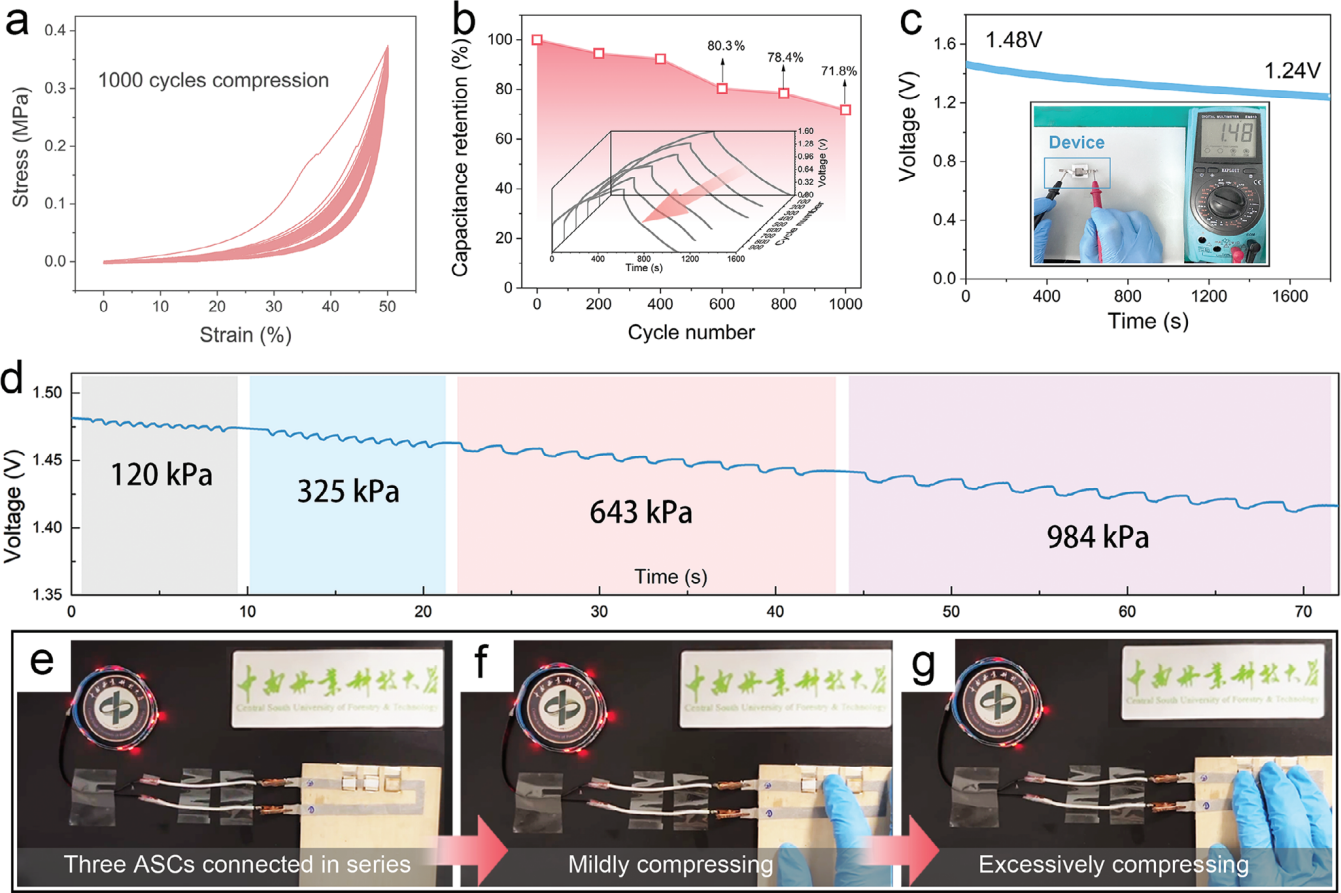
Electrochemical stability of the ASC under compressive conditions. a) *σ–ε* curves showing fatigue resistance at 50% strain for 1000 cycles. b) Retention of original capacitance during 1000 cycles compression. The inset presents GCD curves of the ASC at a current density of 4 mA cm^−2^ recorded during the compression tests. c,d) The *v*–*t* curves of the ASC device recorded at (c) the original state and (d) under various compressive conditions. e–g) Photographs demonstrating three ASC devices powering a series of LEDs under different compressive conditions.

The electrochemical stability of the ASC after cyclic compression was evaluated. As shown in **Figure** **6**a, the ASC demonstrated remarkable resilience, enduring 1000 cycles of compression at 50% strain while retaining a high stress level of 79.6%. This observation underscores its robust structural integrity. Approximately 80.3% of the initial capacitance was preserved after 600 cycles under 50% strain (Figure 6b). Even when underwent 1000 cycles, the ASC still retained 71.8% of its original capacitance, indicative of outstanding cyclic stability under high compression. Further investigation into the electrochemical stability of the ASC device under practical operating conditions was conducted. As shown in Figure 6c, there is no significant leakage observed in a fully charged ASC under ambient conditions and its original state with a mere 0.24 voltage loss recorded within an hour, which is an essential prerequisite for the utilization of the device in compressible SCs applications. Figure 6d displays the voltage variation curve of a single fully charged ASC device. It is evident that consecutive application of pressure ranging from 120 to 984 kPa induces negligible voltage response in the device. Moreover, three ASC devices connected in series were employed to illuminate an LED strip (Figure 6e). When moderate pressure was applied to one of the devices with a finger, there was no discernible change in LED brightness (Figure 6f). Even under excessive stress applied simultaneously to all three devices, the LED continued to operate normally (Figure 6g), indicative of exceptional compression resistance of the WNCS||NiCo ASC. Consequently, the outstanding compressive electrochemical stability of the WNCS||NiCo ASC makes it ideal for diverse applications, including flexible electronics integration and powering devices prone to high impact and compression.

## 3 Conclusion

In summary, we have successfully developed a 3D elastic WNCS electrode with a wrinkled nano‐lamellar structure by a facile “top–down” construction strategy for high‐performance compressible SCs. Specifically, the multilayer nanocarbon sheets, derived from delignification and carbonization of wood, impart the electrode with a higher graphitization degree, significantly increased SSA of 601.7 m^2^ g^−1^, and more abundant meso‐ and micropores compared to conventional wood carbon. The stacking and interlocking of such wrinkled nanolamellae imbue the WNCS with highly compressive properties (580.6 kPa stress at reversible 70% strain) and fatigue durability (1000 cycles at 60% strain). With a thickness of 3.6 mm, the WNCS electrode exhibits a high specific areal capacitance of 4.21 F cm^−2^ at 1 mA cm^−2^. Even under an ultrahigh current density of 100 mA cm^−2^, the electrode retains 56.7% of its capacitance compared to that at 1 mA cm^−2^. Moreover, the 3D compressible ASC, assembled using WNCS as the anode and NiCo LDH/CF as the cathode, demonstrates exceptional mechanical elasticity and electrochemical performance. These include a high stress strength of 1.03 Mpa at a strain of 60%, 1000 cycles of compression at 50% strain, an ultra‐high specific area capacitance of 1.79 F cm^−2^, and a maximum energy density of 607.1 µWh cm^−2^. Importantly, the ASC exhibits high electric robustness in the absence of additional protective housing, maintaining 71.9% of its original capacitance after 1000 cycles of repeatedly load under 50% strain and being able to withstand repeated loads in a wide stress range of 0–984 kPa. These results could tackle the low stress tolerance limits of previously reported compressible SCs. Such sustainable, low‐cost, and scalable elastic electrode materials show potential advantages to be used to develop various ameliorative hybrid electrodes for other types of compressible energy devices.

## 4 Experimental Section

### Materials

Balsa wood was purchased from Ji'an Jinhong New Material Co. Ltd. Sodium chlorite (NaClO_2_, 80%) was obtained from Shanghai Macklin Biochemical Co. Ltd. Sodium hydroxide (NaOH), potassium hydroxide (KOH), cobalt nitrate Co(NO₃)₂·6H₂O, nickel nitrate Ni(NO₃)₂·6H₂O, acetic acid, ethanol, tertbutyl alcohol (TBA), and polyvinyl alcohol (PVA) were purchased from Sinopharm Chemical Reagent Co. Ltd. All reagents were used without further purification.

### Preparation of Wood Sponge

The wood sponge is prepared by chemical treatment to selectively remove lignin and hemicellulose from the wood cell wall. Specifically, 500 mL of 2 wt% NaClO_2_ aqueous solution was prepared, and the solution pH was adjusted to 4.6 by acetic acid. Eight pieces of size 2 × 2 × 2 cm of balsa wood were immersed in the solution by vacuum impregnation, and heated to 100 °C for 6 h in a water bath to remove lignin. Subsequently, the wood was treated with an 8 wt% NaOH solution at 80 °C for 8 h to remove the hemicellulose fraction. To ensure the complete removal of lignin, the wood was treated again with a NaClO_2_ aqueous solution. The treated samples were washed carefully in ethanol and deionized water to remove the residual chemical substances, and then were replaced with TBA and freeze‐dried to obtain a wood sponge.

### Preparation of WNCS

The wood sponge was placed in a tubular furnace (BTF‐1400C‐II, Anhui Beiyike Equipment Technology Co., Ltd.), and nitrogen (99.999% purity) was fed with a flow rate of 200 sccm. The furnace was heated to 1000 °C with a heating rate of 10 °C to pyrolyze the wood for 4 h. Afterward, the sample was naturally cooled down to room temperature under nitrogen protection, and then acid‐washed with a 10 wt% nitric acid solution at 120 °C to improve its wettability. Finally, WNCS was obtained by drying at 60 °C.

### Synthesis of NiCo DHL/CFs Anode

NiCo LDH was prepared on the surface of CFs by electrodeposition. Specifically, 2 mm Co(NO₃)₂·6H₂O and 1 mm Ni(NO₃)₂·6H₂O were dissolved in 50 mL deionized water as the electrolyte. Biomass carbon fibers (CFs) were prepared as a good substrate for electrodeposition according to the method reported in the previous work.^[^ ^1^ ^]^ In the electrochemical workstation, using clean CFs as the working electrode, Ag/AgCl as the reference electrode, and platinum sheet as the counter electrode, a 5‐cycle CV test was performed at 5 mV s^−1^, −1.2–0.2 V. After the reaction, the sample was washed with deionized water and ethanol, and vacuum dried at 60 °C for 12 h to obtain the NiCo LDH/CFs cathode.

### Assembly of WNCS||NiCo ASC

Asymmetric solid–state supercapacitors were assembled using layered elastic WNCS as the anode, NiCo DHL/CFs as the cathode, cellulose paper as the separator, and PVA/KOH as the electrolyte. First, 5 g of PVA was dissolved in 45 g of deionized water at 100 °C, and then the electrode materials and separator were immersed in the PVA solution. Bubbles were removed by vacuum filtration. The impregnated PVA material was assembled in a sandwich structure (i.e., positive electrode/separator/negative electrode), and then dried at 60 °C to completely evaporate the water in the PVA. Finally, the assembled device was immersed in a 3 m KOH solution for 24 h to fill the PVA with the electrolyte.

### Materials Characterization

The micro‐ and nanostructures and morphologies of materials were characterized by field‐emission scanning electron microscopy (SEM, Zeiss Gemini 300) and transmission electron microscopy (TEM, FEI Talos F200X). Energy dispersive X‐ray spectroscopy (EDS) was used to analyze elemental components and the distribution of the material. Pore structure and Brunauer–Emmett–Teller (BET) SSA were analyzed by N_2_ adsorption–desorption tests by an accelerated surface area and porosimeter system (Micromeritics, TriStar II 3flex). The chemical composites and functional groups of Natural wood and wood sponge were determined using Fourier Transform Infrared (FT‐IR, Bruker Vertex 70) spectroscopy at the scan rate of 500–4000 cm^−1^. The crystalline structure of the carbonaceous materials was characterized by an XRD diffractometer (Bruker D8 Advance TXS) at a scan rate (2q) of 5 min^−1^ and a scan range from 5° to 90°. Raman spectra were recorded using a Renishaw InViamicro‐Raman system with an excitation wavelength of 514 nm. The compression tests of materials were performed on an electronic universal testing machine (SANS, CMT6103).

### Electrochemical Measurements

Electrical conductivity was tested on four‐point probe working stationel (ST2258C, Shanghai Gejing Semiconductor Co., Ltd, China). Cyclic voltammetry (CV), galvanostatic charge–discharge (GCD), and electrochemical impedance spectroscopy (EIS) tests of electrode materials and ASC devices were conducted on a multichannel electrochemical workstation (CS310X, Wuhan CorrTest Instruments Co., Ltd, China). EIS was carried out at open circuit potential in a frequency range from 0.05 Hz to 40 kHz with a perturbation of 10 mV. The specific area, volumetric, and gravimetric capacitances are calculated using the following equations based on GCD curves:

(1) $$C_{a}=\frac{I\times \Delta t}{\Delta V\times a}$$

where *C*_a_ represents the specific areal (*C*_s_, cm^2^), gravimetric (*C*_m_, mg), or volumetric (*C*_v_, cm^3^) capacitances. Accordingly, *a* refers to area, mass, or volume. *I*, Δ*t*, and Δ*V* are the current density (mA), discharge time (s), and potential window or voltage range (V) excluding the potential drop, respectively.

The specific energy density and specific power density of ASC were calculated based on the following equations:

(2) $$E=\frac{1}{2}C_{a}{\left(\Delta V\right)}^{2}$$

(3) $$P=\frac{E}{\Delta t}$$

where *E* is the energy density (µWh cm^−2^, mWh cm^−3^, or Wh kg^−1^), *C* is the specific capacitance, Δ*V* is the voltage range (V) excluding the potential drop, *P* refers to power density (mW cm^−2^, mW cm^−3^, W kg^−1^).

## Conflict of Interest

The authors declare no conflict of interest.

## Supporting information



Supporting Information

## Data Availability

The data that support the findings of this study are available from the corresponding author upon reasonable request.;
